# Efficacy in the reduction ratios of middle molecules with the use of medium cut-off dialyzers and reduced dialysate flows: a cohort study

**DOI:** 10.3389/fmed.2025.1663750

**Published:** 2025-10-09

**Authors:** Juan C. Castillo, Cesar Doria, Javier Cely, David Camargo, Viviana Orozco, Daniel Ducuara, Jasmin Vesga, Mauricio Sanabria, Angela Rivera, Bengt Lindholm, Peter Rutherford

**Affiliations:** ^1^Renal Care Services Agencia Soacha, Bogotá, Colombia; ^2^Renal Care Services Sucursal Bucaramanga, Bucaramanga, Colombia; ^3^Renal Care Services Agencia Nacional, Bogotá, Colombia; ^4^Renal Care Services Agencia Instituto Nacional del Riñon, Bogotá, Colombia; ^5^Renal Care Services Agencia Cardioinfantil, Bogotá, Colombia; ^6^Renal Care Services Agencia San Rafael, Bogotá, Colombia; ^7^Renal Care Services Colombia, Bucaramanga, Colombia; ^8^Renal Care Services Latin America, Bogotá, Colombia; ^9^Vantive, Deerfield, IL, United States; ^10^Renal Medicine, Karolinska Institutet, Stockholm, Sweden; ^11^Vantive, Zurich, Switzerland

**Keywords:** hemodialysis, high flux hemodialysis, expanded hemodialysis (HDx) therapy, green dialysis, theranova dialyzer

## Abstract

**Introduction:**

Expanded hemodialysis (HDx) enabled by Theranova increases the clearance of medium-sized molecules, improving clinical outcomes such as hospitalization and mortality. The objective of the study was to compare solute reduction ratios of medium-molecular-weight uremic toxins by HDx versus high-flux hemodialysis (HF-HD) with dialysate flow rates of 400 mL/min and 500 mL/min.

**Methods:**

In 287 prevalent adult dialysis patients (mean age 61 years, 67% were men, 42.5% had diabetic kidney disease, and 16.7% had urine output ≥250 mL/day), the solute reduction ratio of circulating middle molecules was determined at 4 weeks and 12 weeks of follow-up in two cohorts, one with HDx (*n* = 137) and one with HF-HD (*n* = 150). A mixed-effects repeated measures model was used to evaluate differences between treatment groups. The frequencies of serious adverse events and hospitalization were also calculated.

**Results:**

The HDx group achieved greater efficiency compared with HF-HD group in removing β2-microglobulin and free light chains (lambda and kappa); this superiority was statistically significant for both dialysate flow rates of 400 mL/min and 500 mL/min. We observed 25.9% fewer serious adverse events in the HDx cohort, none of which were causally related to the Theranova dialyzer or HF-HD treatment during 11,409 sessions.

**Conclusion:**

HDx enabled by Theranova dialyzer significantly improved the removal of medium-molecular-weight uremic toxins compared to HF-HD at dialysate flow rates of 500 mL/min and 400 ml/min, with fewer serious adverse events and hospitalization events. These findings support the use of the environmentally sustainable dialysis treatment of HDx with dialysate flow rate of 400 ml/min.

## Introduction

Since the beginning of hemodialysis therapy, one of the therapeutic objectives has been the clearance of so-called uremic toxins that cannot be eliminated by failing kidneys. Throughout these luminous years, there has been a remarkable understanding of the role played by uremic solutes and their relationship to the membranes used for hemodialysis (1). More than two decades ago, the HEMO study shed light on the importance of improving the clearance of medium-sized molecules in hemodialysis (2). Increased clearance of medium-sized molecules provided by high flux membranes has been documented in efficacy studies (3) and in studies of effectiveness outcomes such as cardiovascular mortality (4). This journey of improvement in the clearance capacities of uremic toxins had a turning point with the advent of medium cut-off membranes that have demonstrated a notable increase in the clearance of larger medium-sized molecules (5–7), and therefore expands the capabilities of hemodialysis, improving symptoms and showing improvements in effectiveness outcomes (8).

In parallel with these developments, the dialysis community has been pursuing more eco-sustainable and planet-friendly models, especially regarding water consumption per hemodialysis session (9, 10). Some groups have advocated for water-sparing strategies including a decrease in dialysate flow (Qd), particularly in patients with low to medium body surface area (11), provided that this is possible without compromising the amount of dialysis provided.

A study using a model to predict optimal dialysate flow for dialysate flows (Qd) of 400, 500 and 700 mL/min, and blood flow (Qb) of 300 mL/min, showed no statistically significant difference in terms of KT delivered or in the proportion of patients reaching a threshold of KT/V > 1.2 per session, which was 100% in all three groups (12).

In the context of expanded hemodialysis (HDx), these experiences open a window of opportunity to search for a dialysis procedure that saves water consumption while maintaining increased clearance capacity of medium-sized molecules. The objective of the present study is to analyze and compare the solute reduction ratios of circulating medium-sized/middle molecules in two cohorts, one treated with HDx and the other with high flux hemodialysis (HF-HD), with dialysate flow rates of 400 mL/min and 500 mL/min.

## Methods

### Study design and population

This is a prospective, observational, analytical, multicenter cohort study of prevalent patients undergoing chronic hemodialysis, defined as receiving hemodialysis (HD) for at least 90 days. From April 1, 2024, patients received dialysis treatment at clinical centers belonging to the Renal Care Services network in Colombia and were followed for up to 12 weeks. Inclusion criteria included being over 18 years old, having prevalent HD, having a minimum session duration of 4 h, having a frequency of three times per week, and having a vascular access device, such as an arteriovenous fistula or graft. Exclusion criteria included patients who did not give informed consent to participate in the study; pregnant women; patients with high comorbidity, as measured by a Charlson Comorbidity Index score greater than or equal to eight; patients with a life expectancy of less than 6 months; and patients with metastatic disease. Two cohorts were assembled: HDx enabled by the Theranova dialyzer and HF-HD. Censored events included kidney transplantation, loss to follow-up, discontinuation of dialysis, change of dialysis provider, change of dialysis modality, change of membrane type (more than 13 consecutive sessions with a membrane change), and recovery of renal function. Stratified random sampling with replacement was used for this study. Patients were stratified at each of the six dialysis clinics according to dialyzer type (Theranova or high flux [HF]). The study protocol was approved by the Cardioinfantil Foundation’s clinical research ethics committee on February 7, 2024 (Minute, Item Number 004) and was registered with the ISRCTN Registry (BIOMED Central) as ISRCTN21098097^1^.

### Data collection

We assessed demographic and clinical characteristics at baseline and every 4 weeks. These included age, sex, ethnicity, CKD etiology, dialysis vintage, Charlson comorbidity index score, Karnofsky performance status score, and history of cardiovascular disease or diabetes. We also recorded body mass index, body surface area, urine output, and serum levels of hemoglobin, phosphorus, albumin (Bromocresol Green method), pre- and post-dialysis urea nitrogen (BUN), high-sensitivity C-reactive protein (hs-CRP), parathyroid hormone (PTH), and Kt/V single pool. Additionally, data on vascular access, dialysis flow rate, ultrafiltration, and blood flow rate were collected. All data was obtained from the Versia^®^ electronic medical record system of Renal Care Services. An internal audit was conducted as part of a data quality assurance process.

### Study outcomes

The primary objective of the study was to compare the effectiveness of dialysis membranes in terms of solute removal by calculating the solute reduction ratios of circulating middle molecules, such as beta-2-microglobulin, kappa and lambda free light chains, and leptin, measured before and after a session of HDx or HF-HD in the two cohorts (HDx or HF-HD) during weeks 4 and 12. For calculations of solute reduction ratios or percent changes, the post-dialysis concentrations were corrected for hemoconcentration using the formula by Bergström and Wehle (13). We included adverse events as secondary outcomes.

### Statistical analysis

Data are presented as mean and standard deviation (SD) for variables with normal distribution and as median and interquartile range (IQR) for variables with non-normal distribution. Categorical variables are expressed as frequencies and percentages. Baseline differences between groups were compared using Pearson’s χ2 test, and continuous variables were analyzed using Student’s *t*-test or the Mann-Whitney U test. When the data included more than two measurements for a quantitative variable, a mixed-effects repeated measures model was used to evaluate differences between groups. The intent-to-treat full analysis set included all eligible patients. Data imputation was not performed, and an analysis of complete cases was conducted. Stata 16^®^ (StataCorp, 2019. Stata statistical software: Release 16. College Station, TX: StataCorp LLC.) was used for statistical analyses.

## Results

### Baseline characteristics

**FIGURE 1:**
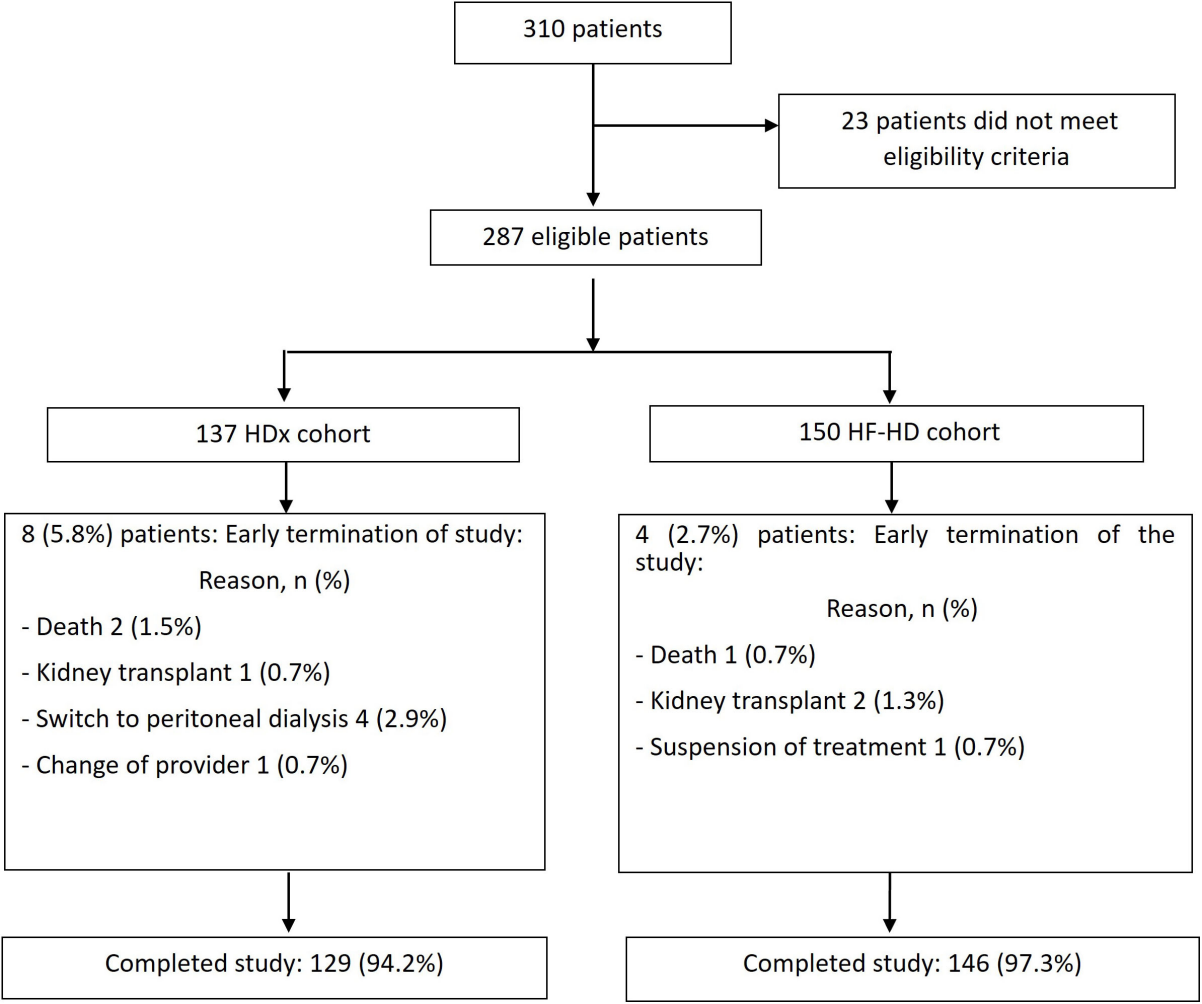
Flowchart of recruitment of patients into the study. Flowchart of the recruitment into the two arms of the study of patients undergoing HDx therapy enabled by Theranova dialyzer (HDx cohort) or those receiving high flux hemodialysis (HF-HD cohort).

A total of 287 patients were eligible, 137 of whom were in the HDx group and 150 in the HF-HD group. After 12 weeks of study follow-up, 97.3% of participants in the HF-HD cohort and 94.2% in the HDx cohort completed follow-up. The main reasons for censoring were dialyzer change (1.4%) and death (1.1%). See Figure 1. At baseline, the mean age was 61.3 years, 67% were men, and the leading causes of CKD were diabetic kidney disease (42.5%), followed by hypertension and glomerular/autoimmune diseases. 22.3% of the patients had a diagnosis of congestive heart failure, and 13.6% had a history of ischemic cardiovascular disease.

The median length of time on dialysis was 5.2 years; 16.7% of the patients still had residual renal function defined as urine output ≥250 mL/day. Details are provided in Table 1.

**TABLE 1:** Demographics and baseline characteristics.

Characteristics	HD-HF	HDx	Full sample	*P*-value^a^
	N = 150	N = 137	N = 287	
Age, years, mean (SD)	62.7 (13.4)	59.7 (14.1)	61.3 (13.8)	0.066
Sex, n (%): male	97 (64.7)	96 (70.1)	193 (67.2)	0.950
Female	53 (35.3)	41 (29.9)	94 (32.8)	
Ethnicity, *n* (%): indigenous	1 (0.6)	0 (0.0)	1 (0.3)	0.286
Afroamerican	4 (2.7)	1 (0.7)	5 (1.7)	
Mestizo	145 (96.7)	136 (99.3)	281 (98.0)	
Diabetes history, *n* (%): Yes	63 (42.0)	59 (43.1)	122 (42.5)	0.855
Congestive heart failure, *n* (%)	27 (18.0)	37 (27.0)	64 (22.3)	0.067
Cerebrovascular event history, *n* (%)	5 (3.3)	2 (1.5)	7 (2.4)	0.304
History of ischemic cardiovascular disease, *n* (%)	20 (13.3)	19 (13.9)	39 (13.6)	0.895
Vintage of KRT, years, median (IQR)	4.6 (2.5; 8.1)	5.6 (3.8; 9.6)	5.2 (2.9; 8.8)	0.008
Charlson comorbidity index, median (IQR)	2 (0; 3)	2 (0; 3)	2 (0; 3)	0.454
Karnofsky scale, mean (SD)	71 (14)	71 (12)	71 (13)	1
Body mass index, kg/m^2^, mean (SD)	25.3 (4.0)	25.5 (4.8)	25.4 (4.4)	0.701
Urine output, ml/day; *n* (%): <250	117 (78.0)	122 (89.0)	239 (83.3)	0.012
≥250	33 (22.0)	15 (11.0)	48 (16.7)	
Hemoglobin, g/dL, mean (SD)	11.3 (1.4)	11.5 (1.6)	11.4 (1.5)	0.091
Albumin, g/dL, mean (SD)	4.1 (0.3)	4.1 (0.3)	4.1 (0.3)	1
Phosphorus, mg/dL, mean (SD)	4.5 (1.2)	5.1 (1.6)	4.8 (1.4)	0.021
C reactive protein, mg/L, mean (SD)	1.0 (3.2)	0.9 (2.9)	1.0 (3.1)	0.506
Kt/V, mean (SD)	1.7 (0.3)	1.7 (0.3)	1.7 (0.3)	1
Urea reduction ratio,% (SD)	75.5 (5.8)	75.1 (6.2)	75.3 (6.0)	0.658
PTHi, pg/dL, median (IQR)	481 (172; 650)	418 (209; 662)	365 (182; 660)	0.331
Membrane type, *n* (%): Dora B-18 (1.8 m^2^)	116 (77.3)	0	116 (40.4)	1
Revaclear 400 (1.8 m^2^)	23 (15.4)	0	23 (8.1)	
Elisio 19H (1.9 m^2^)	11 (7.3)	0	11 (3.8)	
Theranova 400 (1.7 m^2^)	0	137 (100)	137 (47.7)	
Vascular access, *n* (%): Graft	3 (2.0)	3 (1.0)	6 (2.1)	0.265
Arteriovenous fistula	147 (98.0)	134 (99.0)	281 (97.9)	
Dialysate flow, ml/min, mean (SD)	421.7 (42.6)	416 (43.8)	419 (43.3)	0.261
Blood flow, ml/min, mean (SD)	332.4 (42.2)	349.4 (46.0)	340.5 (50.0)	0.001
Body surface area, mean (SD):				
Dialysate flow, 400 ml/min	1.6 (0.2)	1.7 (0.2)	1.7 (0.2)	0.214
Dialysate flow, 500 ml/min	1.9 (0.2)	1.9 (0.2)	1.9 (0.2)	0.885
Session time, hours, mean (SD)	4.0 (0)	4.0 (0)	4.0 (0)	1

*^a^*Statistical difference between HD-HF and HDx: Categorical variables were compared with Pearson’s χ2 test and continuous variables were analyzed with Student’s *t*-test or Mann-Whitney test. KRT, kidney replacement therapy.

### Outcomes

**FIGURE 2:**
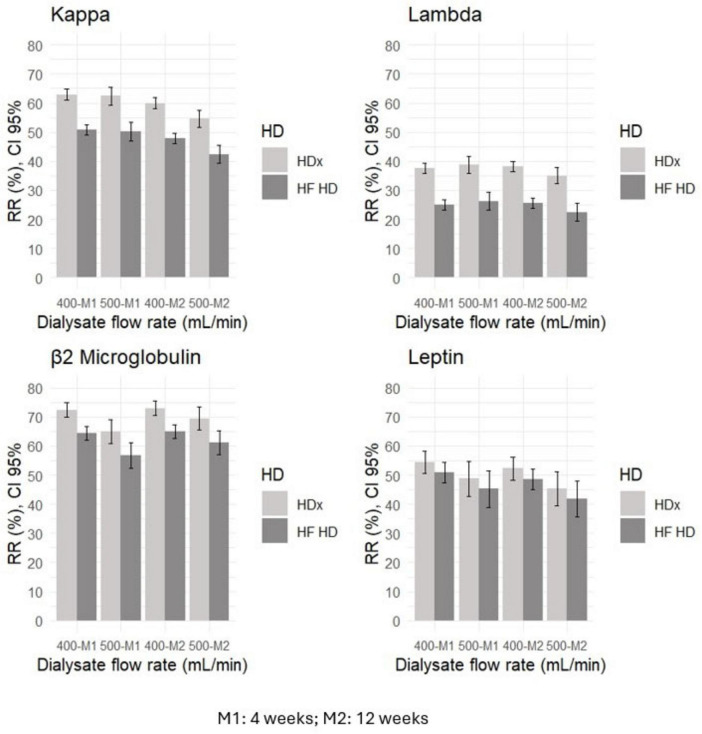
The relationship between dialysate flow rate and reduction ratios (RR) for middle molecules, such as kappa and lambda free light chains, leptin, and β2 microglobulin, in HDx and HF-HD cohorts.

Solute reduction ratios for circulating middle molecules, including kappa and lambda free light chains, beta 2-microglobulin, and leptin, were calculated at week 4 and at week 12. HDx showed significantly greater reduction ratios than HF-HD for kappa and lambda free light chains and for beta 2-microglobulin, indicating greater clearance of these molecules (*p* < 0.01). Although there was a trend toward greater leptin clearance with HDx, the differences were not statistically significant (*p* > 0.05). Details are presented in Table 2. Additionally, we used a mixed-effects repeated measures model to evaluate the effect of confounding variables on reducing middle molecules. No statistically significant differences were observed in the reduction ratios of kappa (*p* = 0.097) and lambda (*p* = 0.521) free light chains, β2-microglobulin (*p* = 0.089), and leptin (*p* = 0.071), when using dialysate flows of 400 or 500 ml/min. Furthermore, we found that the likelihood of achieving an effective reduction in lambda free light chains is 55% greater in the HDx group. The probability is 27% for kappa and 15% for β2-microglobulin, compared to the group treated with HF-HD, as illustrated in Figure 2 and Table 3.

**TABLE 2:** Differences between HF-HD and HDX treatment groups in solute reduction ratios for kappa and lambda free light chains, leptin and β2 microglobulin.

Time	Parameter	HF- HD	HDx	Difference [95% CI]	*P*-value
		Mean [95% CI]	Mean [95% CI]		
4 weeks	Kappa	50.6 [48.8–52.4]	62.9 [60.7–65.1]	−12.3 [−15.1 to −9.4]	<0.001
	Lambda	24.8 [23.0–26.6]	38.2 [36.2–40.2]	−13.4 [−16.1 to −10.8]	<0.001
	Leptin	50.4 [46.5–54.3]	52.6 [48.8–56.4]	−2.1 [−7.6 to −3.3]	0.438
	β2 microglobulin	62.9 [60.1–65.6]	70.9 [68.3–73.5]	−8.1 [−11.8 to −4.2]	<0.001
12 weeks	Kappa	46.8 [45.0–48.7]	58.4 [56.6–60.1]	−11.5 [−14.1 to −8.9]	<0.001
	Lambda	25.4 [23.6–27.2]	36.8 [35.1–38.6]	−11.4 [−13.9 to −8.9]	<0.001
	Leptin	47.1 [43.7–50.6]	50.6 [46.7–54.6]	−3.5 [−8.7 to −1.6]	0.189
	β2 microglobulin	64.6 [62.0–67.2]	71.6 [69.4–73.9]	−7.0 [−10.5 to −3.5]	<0.001

**TABLE 3:** Factors associated with the reduction ratios for middle molecules (lambda and kappa free light chains, β2 microglobulin and leptin). Differences are expressed as relative risk (95% confidence interval, 95%CI).

Middle molecules	Relative risk	95%CI		*P*-value
**Reduction ratio: lambda**				
HDx vs. HF-HD	1.54	1.44	1.66	<0.001
Qd400 mL/min vs. 500 mL/min	0.97	0.89	1.06	0.521
Blood flow, mL/min	1.00	0.99	1.01	0.360
Urine output ≥250 mL/day	0.94	0.85	1.03	0.173
Vintage of KRT, years	1.00	0.99	1.01	0.087
Body mass index, kg/m^2^	0.99	0.98	1.00	0.082
**Reduction ratio: kappa**				
HDx vs. HF-HD	1.27	1.2	1.33	<0.001
Qd400 mL/min vs. 500 mL/min	1.06	0.99	1.13	0.097
Blood flow, mL/min	1.00	0.99	1.01	0.165
Urine output ≥250 mL/day	0.92	0.85	0.98	0.016
Vintage of KRT, years	1.00	0.99	1.01	0.370
Body mass index, kg/m^2^	1.00	0.99	1.01	0.255
**Reduction ratio: β2** **microglobulin**				
HDx vs. HF-HD	1.16	1.09	1.23	<0.001
Qd400 mL/min vs. 500 mL/min	1.07	0.99	1.15	0.089
Blood flow, mL/min	1.00	0.99	1.01	0.253
Urine output ≥250 mL/day	1.01	0.85	1.09	0.919
Vintage of KRT, years	1.00	0.99	1.01	0.465
Body mass index, kg/m^2^	1.00	0.99	1.01	0.443
**Reduction ratio: leptin**				
HDx vs. HF-HD	1.07	0.94	1.19	0.322
Qd 400 mL/min vs. 500 mL/min	1.14	0.98	1.33	0.071
Blood flow, mL/min	1.00	0.99	1.01	0.905
Urine output ≥250 mL/day	0.92	0.78	1.07	0.294
Vintage of KRT, years	1.00	0.99	1.01	0.949
Body mass index, kg/m^2^	0.98	0.97	1.00	0.017

A dialysate flow rate of 500 ml/min was prescribed for 57 patients, while the remaining 230 patients were prescribed a flow rate of 400 ml/min. Qd, dialysate flow rate; KRT, kidney replacement therapy.

**FIGURE 3:**
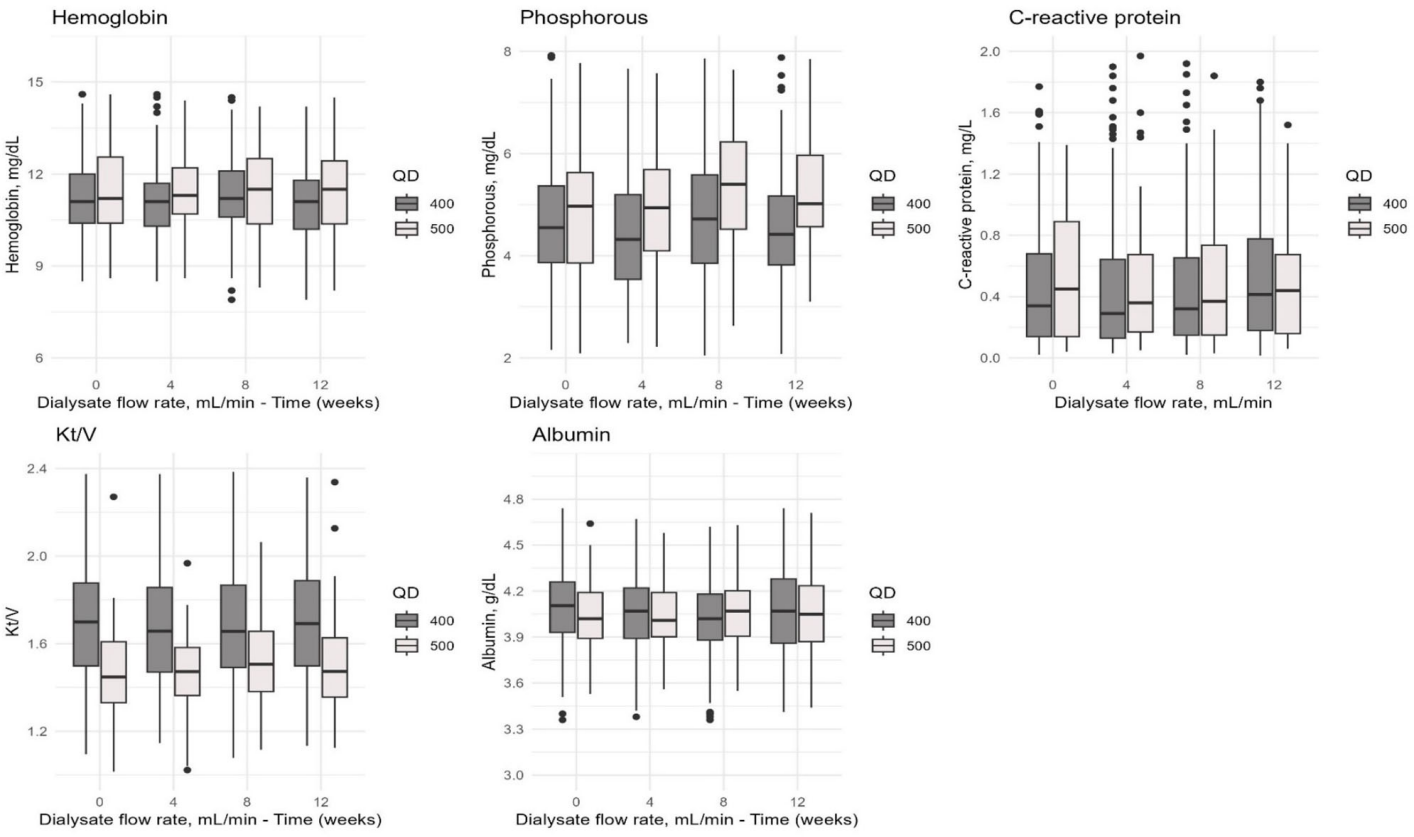
The distribution of hemoglobin, phosphorus, C-reactive protein, Kt/V, and albumin values in the combined cohort of patients undergoing HDx or HF-HD with dialysate flow rates of 400 and 500 mL/min. Measurements were taken at different time points (0, 4, 8, and 12 weeks) to evaluate how these values changed over time.

We observed a trend toward improved levels of hemoglobin, phosphorus, Kt/V, parathyroid hormone, and high-sensitivity C-reactive protein in the HDx group compared to the HF-HD group over time. However, these differences were not statistically significant (*p* > 0.05). We observed a similar pattern over time in both cohorts regarding albumin. See Figure 3 for details.

In terms of safety, a total of 53 adverse events were recorded during the follow-up period. The most frequent causes were cardiovascular disease (18.9%), cerebrovascular disease (17%), gastrointestinal disease (13.2%), and trauma (13.2%). Details are presented in Table 4. Regarding serious adverse events, we observed that the HDx group had a 50% lower frequency compared to the HF-HD group (75.9%). This difference was statistically significant (*p* = 0.048). Additionally, we found that the HDx group had a lower hospitalization rate (6.6%) than the HF-HD group (13.3%). This difference was also statistically significant (*p* = 0.028). Of the total serious and non-serious adverse events, none were causally related to the use of any type of dialyzer.

**TABLE 4:** Number and distribution of adverse events in HF-HD (*n* = 146) and HDx (*n* = 129) cohorts.

Adverse event	HF-HD	HDx	Total
Cardio-cerebrovascular	5 (17.2)	5 (20.8)	10 (18.9)
Other vascular disorders	4 (13.8)	5 (20.8)	9 (17.0)
Digestive diseases	6 (20.7)	1 (4.2)	7 (13.2)
Traumas	3 (10.3)	4 (16.7)	7 (13.2)
Bacteremia/septicemia/ infections	3 (10.3)	2 (8.3)	5 (9.4)
Others	1 (3.5)	3 (12.5)	4 (7.6)
Non-vascular nervous disease	1 (3.5)	1 (4.2)	2 (3.8)
Tumors or neoplasms	1 (3.5)	1 (4.2)	2 (3.8)
Genito-urinary disease	1 (3.5)	0.0	1 (1.9)
Mental disorders	1 (3.5)	0.0	1 (1.9)
Respiratory disease	1 (3.5)	0.0	1 (1.9)
Hematopoietic disease	0	1 (4.2)	1 (1.9)
Musculoskeletal system disease	0	1 (4.2)	1 (1.9)
Endocrine/metabolic disease	1 (3.5)	0.0	1 (1.9)
Skin and subcutaneous tissue	1 (3.5)	0.0	1 (1.9)
Total	29 (100)	24 (100)	53 (100)

**FIGURE 4:**
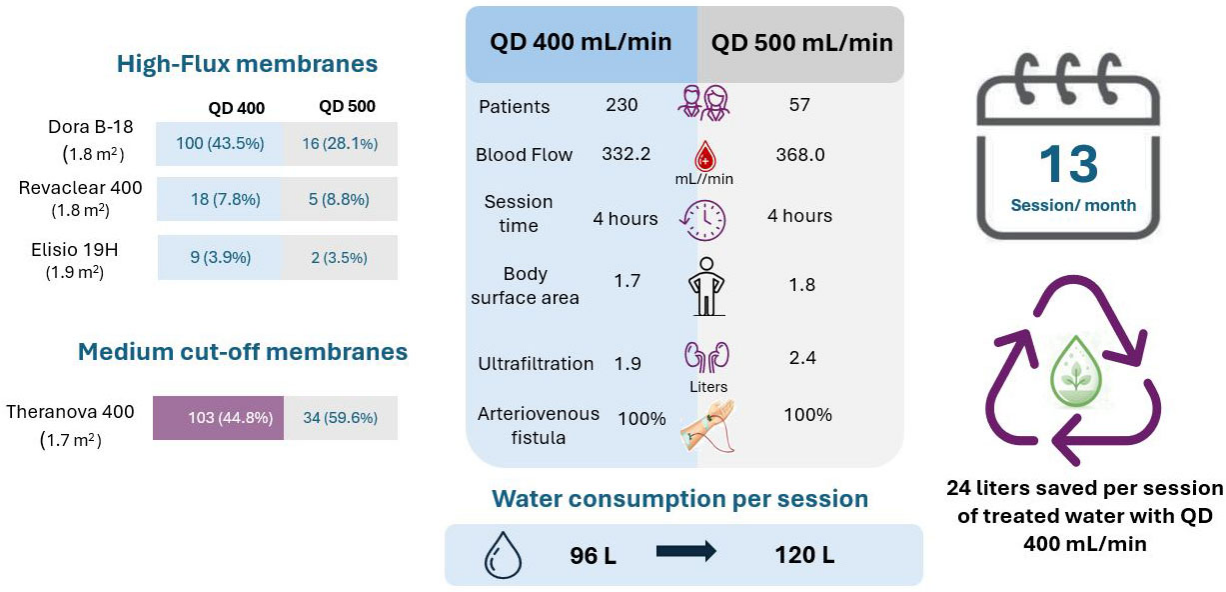
Characteristics of hemodialysis prescription according to dialysate flow rate (Qd 400 vs. Qd 500) and water consumption per session.

In addition, a hemodialysis prescription with a Qd of 400 ml/min uses an average of 96 liters of treated water per 4-h session, which compared to an average of 120 liters per session with a Qd of 500 ml/min represents water savings of 24 liters per session per patient. Figure 4 shows the details of patient prescriptions and water savings according to the Qd used.

## Discussion

The last decade has seen a growing body of evidence regarding the efficacy of medium cut-off membranes in terms of clearance of a wide range of medium-sized molecules as well as the safety of these novel dialyzers (14–16), aspects that have been corroborated in integrative research with meta-analysis (17, 18). In this same sense, the present study found that the reduction ratio of large medium-sized molecules (kappa and lambda free light chains and β2-microglobulin) was higher with medium cut-off membranes when compared with high-flux membranes.

Furthermore, a point that constitutes a novel result of this study is that these increased clearance capacities of large medium-sized molecules provided by the medium cut-off dialyzers are maintained also when a dialysate flow of around 400 mL/min is used, thus opening a window of opportunity for the use of the more eco-sustainable blood purification method of expanded hemodialysis (HDx), potentially allowing substantial water savings.

The possibilities of decreasing dialysate flow without compromising the clearance of low molecular weight molecules have already been reported (11, 12), and the present study shows that it is plausible to decrease the dialysate flow rate in patients treated with HDx without compromising the reduction ratio of medium-sized molecules. In terms of high flux dialyzers, it has been observed that it is possible to reduce dialysate flow rates without affecting the efficiency of the hemodialysis procedure if the Qb to Qd ratio remains in the range of 1–1.5 to 1–2 (19, 20). The present study shows that the clearance capacity of these novel medium cut-off membranes allow this to be equally valid for medium-sized molecules between 10,000 and 45,000 Da.

The use of a mixed model of repeated measurements where the dependent variables are the reduction ratio values of medium molecules, showed that these values are higher in patients dialyzed with medium cut-off membranes when compared with HF-HD, and that in addition the use of decreased levels of dialysate flow does not affect the reduction ratio values when compared with dialysate flows of the order of 500 mL/min. These results had already been suggested by a study in Japan in HD patients, where the decrease in dialysate flow to 400 mL/min did not compromise the clearance of β2-microglobulin, even when prescribing blood pump flow in a 1:2 ratio with the dialysate flow (21). In fact, usual practice recommends that the optimal dialysate flow rate (Qd) be 1.5–2.0 times the blood flow rate (Qb) (22); however, our results are being reported with a Qd of around 1.3 times the Qb, without affecting the reduction ratios of small or medium molecules.

It is worth highlighting that from the dialyzer safety point of view, during the follow-up period, there were no serious or non-serious adverse events causally related to the use of either medium cut-off or high-flux membranes, an aspect that had already been observed in other reports (8, 11, 14, 23).

In addition, prescribing a dialysate flow rate has clinical and sustainability implications. Using a dialysate flow rate of 400 mL/min instead of 500 mL/min can generate substantial water savings. Projecting this savings of 24 liters of treated water per session to 1,000 patients yields a cumulative savings of 3,744,000 liters per year. From a public health perspective, this volume is equivalent to the annual domestic water consumption of 85 people, assuming an average daily usage of 120 liters per person.

The strengths of this study encompass the prospective cohort design involving a network of renal clinics, utilizing random sampling with replacement. This methodology facilitates the analysis of a subpopulation that more accurately reflects the original population. Additionally, the primary outcome of interest, reduction ratios of medium-sized molecules, is predominantly affected by variables related to the dialysis procedure, such as session time, Qb, and Qd, rather than by variables associated with clinical history, sociodemographic factors, anthropometric characteristics, or residual kidney function.

Among the limitations of the study, it should be noted that, due to the study design and the limited follow-up duration, it is not feasible to assess effectiveness outcomes such as survival rates, hospitalization events, or quality of life. Furthermore, as this is an observational study, there is always the potential for bias, which in this case would have little effect on the outcome of interest (reduction ratios of medium-sized molecules), considering that this outcome is fundamentally related to the duration of the dialysis session, the type of dialysis membrane used and the blood pump flow (Qb) and dialysate flow (Qd), and not to other baseline variables.

## Conclusion

Solute reduction ratios of circulating middle molecules were determined in two cohorts of dialysis patients, one with HDx and one with HF-HD, at 4 weeks and 12 weeks of follow-up, and with dialysate flow rates of 400 mL/min and 500 mL/min. The main finding is that use of HDx therapy, enabled by the Theranova dialyzer, was associated with a significant improvement in removing medium-molecular-weight uremic toxins when compared to HF-HD. This improvement was noted at 500 mL/min and 400 mL/min dialysate flow rates. Additionally, HDx therapy resulted in fewer serious adverse events and a reduced number of hospitalizations. These findings encourage the dialysis community to pursue the more eco-friendly dialysis treatment option of HDx, potentially allowing substantial water savings.

## Data Availability

The raw data supporting the conclusions of this article will be made available by the authors, without undue reservation.

## Footnotes

1
https://doi.org/10.1186/ISRCTN21098097
